# Security lies in obedience - Voices of young women of a slum in Pakistan

**DOI:** 10.1186/1471-2458-10-164

**Published:** 2010-03-26

**Authors:** Saima Hamid, Eva Johansson, Birgitta Rubenson

**Affiliations:** 1 Global Health, Department of Public Health Sciences, Karolinska Institute, Stockholm, Sweden; 2 Health Services Academy, Ministry of Health, Government of Pakistan, Islamabad, Pakistan; 3 Nordic School of Public Health, Gothenburg, Sweden

## Abstract

**Background:**

Existing literature shows that young people, especially women, have poor knowledge about sexuality and reproductive health. Many of the difficulties young women experience are related to beliefs and expectations in society making them more vulnerable to reproductive ill health. The objective of this study was to explore how young women living in a slum in Islamabad are prepared for marriage and how they understand and perceive their transition to marriage and the start of sexual and childbearing activity.

**Methods:**

Twenty qualitative interviews and three focus group discussions were conducted with young women residing in a slum of Islamabad. Content analysis was used to explore how the participants represented and explained their situation and how decisions about their marriage were made.

**Results:**

The main theme identified was *security lies in obedience*. The two sub-themes contributing to the main theme were *socialization into submissiveness *and *transition into adulthood in silence*. The theme and the sub-themes illustrate the situation of young women in a poor setting in Pakistan.

**Conclusion:**

The study demonstrates how, in a culture of silence around sexuality, young women's socialization into submissiveness lays the foundation for the lack of control over the future reproductive health that they experience.

## Background

Although teenage marriages are on the decline in Pakistan, one out of six women aged 15-19 years is married [1]. Strong societal, cultural and religious expectations are attached to the sexual innocence and ignorance of women as a sign of purity and virginity, with marriage marking the beginning of sexual relations and childbearing [2]. There is great societal pressure on parents to arrange marriages for their daughters [2], with marriages traditionally arranged by families with minimal involvement of the couple [3]. In the 2002 Adolescent and Youth Survey of Pakistan, 80% of women and 85% of men reported being married to relatives [4]. Marriage initiates new living arrangements and many new relationships, including the husband and his family, and most women find motherhood the main focus of their new life at the expense of personal or relationship development in other areas [3]. Existing literature shows that young people, especially women, have poor knowledge about sexuality and reproductive health [5-10]. A community-based study by Sajan & Fikree (2002) in the squatter settlements of Karachi found a high prevalence of gynaecological morbidity among young married women. Women who began sexual activity in their teens, as compared to women who started after 25 years of age, reported a greater burden of reproductive ill-health. This affirms the risks associated with early marriage and the need to improve and broaden reproductive health services and education [11]. In an earlier study the authors interviewed newly married young women in the same slum area about their experiences of marriage. A narrative analysis of the interviews revealed the submissive nature of the respondents [12]. The submission described by participants was instilled in the young women through the impact of diverse levels of the family, community and society on their lives: their parents deciding about their marriage, often without their consent, the extensive demands placed on them by their parents-in-law and the pervasive societal expectations for them to be obedient in all spheres of life. Many of the difficulties young women experience are related to societal beliefs and expectations that make them more vulnerable to reproductive ill health [11,13,14].

To further investigate the situation of young women living in the slum area in Islamabad this study explores how they are prepared for marriage and perceive their transition to marriage and start of sexual and childbearing activity.

## Methods

A qualitative approach using latent content analysis [15] was used to explore the situation of poor young urban women at the time of their marriage and to study their knowledge of and expectations for married life. In qualitative research human behaviour is understood from the perspective of those being studied; their perceptions, attitudes and experiences are the focus [16]. For this purpose the Principal Investigator (PI) approached the respondents through a community worker and met them multiple times to establish rapport with them. Multiple meetings helped the participants to open up to the PI and discuss sensitive issues regarding sexuality and growing up with reference to their marriage and other related topics of their choice. In this respect unstructured interviews helped elaborate on the topics of participants' choice and probe further their concerns something which could not have been achieved through participatory observations. In using in-depth interviews and focus group discussions (FGDs) information was sought to increase the understanding of young women's interpretations of their situation [16]. The FGDs were conducted following the in-depth interviews using a field guide based on the interviews with the respondents to further explore young women's preparedness for marriage, their knowledge about sexuality, sources of information and their experience of growing up.

### Research Team

The research team comprised a Pakistani medical doctor specialized in public health (PI), a Pakistani community worker and two Swedish public health scientists. As a Pakistani woman and public health doctor the PI had both social and cultural knowledge, spoke the language and could move in the community without arousing curiosity. The community worker was a resident of the community who met regularly with the women's groups and played a key role in giving the insider's perspective to the study and facilitating the entry of the PI to the community. The insider's view of the Pakistani researcher was broadened by the outsider's view of the Swedish researchers, whose experiences living and working in low-income countries enriched the understanding of the data and contributed to the analysis.

### Setting

The study was conducted in a slum community in the outskirts of Islamabad city. There are 900 houses in the community with almost 400 having more than one family living in the house. The residents are mainly daily labourers and the majority are illiterate. Few women have attended school beyond the primary level and many have never even started. The young women have limited opportunities for employment and are mostly married shortly after puberty. Their marriage is viewed as a social and religious duty for the parents. If the marriage is delayed it is usually rather because of economic reasons than for lack of finding a suitable partner. The family has to save or generate resources to bear the expenditure of dowry and wedding celebrations.

### Participants and Data Collection

With the help of the community worker, young women aged 15-24 years and engaged to be married within three months were identified. Twenty women who agreed to participate were included in the study. Parental consent was taken as well. Interviewing was done till saturation was reached. Altogether twelve15-19 year old and eight 20-24 year old women were interviewed. The PI interviewed all of the participants. Given the sensitive issues under discussion the authors counter checked whether the age of the PI could be an obstacle in opening up of the respondents to the PI and candidly sharing concerns about growing up. A younger interviewer could have been more aware of the world of the young women; using familiar language and raising issues with them she could facilitate their communication and discussion with her. For this five of the respondents were also interviewed by a young data collector. Since the information gathered by the data collector and the PI was the same, it was decided that the interviews carried out by the PI were valid. The participants were interviewed in their homes on three occasions with a few days in between to overcome the barrier of talking about sensitive issues with the researcher. This series of conversational interviews gave the women an opportunity to expand on a range of issues that they wanted to discuss [16]. Their knowledge about sexual activity, child bearing and sources of sexual and reproductive health information were explored. Following the interviews, three focus group discussions (FGDs) were held with 14 additional 15-19 year old women. Four or five young women participated in each FGD, which were conducted in the home of one of the participants at their choice. The FGDs were conducted to explore participants' views about adulthood with a focus on womanhood. The young women were asked to base their answers on community perceptions and not on personal feelings [17].

### Data Management and Analysis

Most of the interviews and FGDs were conducted in the local language by the principal investigator (PI) who also transcribed and translated them into English. The transcripts were read several times to gain an in-depth understanding of participants' life experiences and their views on their preparation for and knowledge about sexual and childbearing activity. The data were analyzed by the research team using latent content analysis, with a focus on the description and interpretation of message meanings and concepts. Latent content analysis entails a constant comparison of different parts with a constant attention to the overall individual- and group-level data in a search to achieve an accurate understanding of the underlying meaning [15]. It is only through this careful examination of the women's perspectives that authors were able to understand the situation of the young women in the study. In the interviews and FGDs meaning-units were identified, condensed and then coded. The codes were grouped into categories and abstracted into sub-themes and a main theme, always maintaining the practice of constant comparison during the coding process.

### Trustworthiness

Various assurances were integrated into this study in order to ensure trustworthiness of the data during data collection and analysis. During data collection, information was collected using both in-depth interviews and focus group discussions. The women that participated in the interviews were visited several times to increase interviewer-participant rapport, to provide participants with the opportunity to recapitulate their story, and to provide the interviewer to confirm what had been told and understood. Two interviews and the FGDs were coded by other qualitative researchers who were not involved in the study and compared to the coding done by the research team. Any differences in coding were discussed and a consensus was reached about the final set of codes. The codes generated from the interviews and FGDs were similar, which added to the credibility of the data. The findings were brought back to the community worker for verification.

### Ethical Considerations

Ethical clearance for the study was granted by the Pakistan Medical Research Council and the Karolinska Institute. Verbal consent was taken from the decision maker in the house and the respondent. In most cases the decision maker was the mother who gave consent in the absence of the father. The consent statement, which explained the study objectives and the expectations of the study participants, was read aloud to facilitate their understanding. Study participants were assured of confidentiality.

## Results

The two sub-themes illustrate the ideals, *submissiveness *and *silence*, that the Pakistani women are socialised into from childhood. These were combined into the theme of the study, *security lies in obedience*, thus illuminating the situation of young women in a poor setting in Pakistan. The data are presented starting with the sub-themes and their relation to the categories of analysis and concludes with how they contribute to the main theme (Table 1).

**Table 1:** Analysis Process for moving from Categories to Themes

Category 1	Category II	Category III	Category 1	Category II
Living up to family expectations	Finding security in learning obedience	Level of freedom defined by family	Becoming a woman in silence	Finding cracks in the wall of silence
Sub-theme I **Socialization into submissiveness**			Sub-theme II **Adulthood transition in and into silence**	

Main Theme **Security lies in obedience**				

### Socialization into submissiveness

The first sub-theme refers to the experiences of the young women who described how they were socialised from childhood into submissiveness and obedience. These ideas represent the expected ideals in the social environment of the local society, which underlie the customs guiding the socialisation of girls. The psychology of participants' parents became apparent in the varying degree of freedom and opportunity that different parents allowed their daughters. This socialization into submissiveness was achieved by:

#### Living up to family expectations

The young women described the behaviours, duties and responsibilities they were brought up to fulfil. They understood their primary role to be tending to their household and listed the typical duties such as cooking and cleaning as the main things that gave them personal value. They had agreed not to attend school, instead staying at home out of a sense of duty and thus enabling their siblings (especially brothers) to go to school. They preferred to work at home, looking after everyone else's needs and literally serving as substitutes for their mothers. To be selfless, loyal and possess empathy for the family were important characteristics valued in their upbringing.

*"My mother is away visiting relatives for the past 5 days. She told me to be a good daughter and look after the family. I have been looking after my younger brothers and sisters and my father and have done rather well. My younger brothers and sisters are happy and not missing Ami (mother) ...." *(15 years old)

#### Finding security in learning obedience

The young women were expected to be obedient and faithful, to defer to their parents for decision-making and to oblige their future in-laws in the same manner. All decisions regarding their marriage were entrusted to the family and the family's (not the women's) opinions were what mattered most.

*"There is a girl, who married by her own choice against her parents will. ...the husband does not work and beats her.... she used to be so pretty and now she is in such a pathetic state. I think she should have married the person her parents wanted her to marry." *(15 years old)

When asked to express their aspirations for the future they were unable to enlist any wishes and showed reservations to elaborate. Disobedience by participants resulted in displeasure on the part of their parents, especially mothers, who stressed that their behaviour reflected their upbringing and thus disobedience reflected badly on the parents. They learnt that the sole path to being looked after and feeling secure was to abide by the rules. The young women said that they had to learn to control their tongue and exercise *sabar *(patience), the main competencies for a successful future married life.

*"My mother tells me to show sabar (patience) and not to answer back. My behaviour is a reflection of my parental upbringing." *(19 years old)

Young female participants illustrated the subservient role of women in society by referring to television serials. A young woman in a FGD referred to a serial that showed how all decision-making lay in the hands of the husband and then concluded that the woman had no control over her life. This perspective was echoed by other participants, who voiced the importance of obeying their future husbands to avoid the consequences of divorce and being left for another wife. They understood the fragility of marriage and the ease of remarriage for a man in society.

#### Level of freedom defined by family

The young women stated that their mobility both inside and outside the home was closely monitored by the elders in the family. They feared that these restrictions would continue after marriage.

*"My fiancé is shakki (does not trust). In the village when we visit them, he does not like it if I talk to our other relatives. I am afraid of what will happen after my marriage."*(19 years old)

### Adulthood transition in and into silence

In the FGDs the young women discussed the challenges of menstruation and body changes, which caused discomfort and raised questions to which they were given few answers. They saw the onset of menstruation as the first sign of growing up and the end of childhood and freedom. They also considered this physiological change as marking the beginning of an era of confinement.

#### Becoming a woman in silence

Not prepared for their first menstruation, participants were shocked by the experience. When they approached their mothers they were told not to talk about it and only shown how to handle the bleeding. Through friends, aunts or their mothers they learnt that they were not to offer prayers during menstruation and to take a cleansing bath once the bleeding stopped. Young women felt severely inhibited in their ability to ask questions about physical and related changes and they understood that keeping silent on women's health issues was part of being a grown-up woman.

*"I stopped going to school as I was afraid of having to deal with menstruation in school. No one at home asked me why I stopped and nobody at home told me to go to school either." *(17 years old)

Silence around sexuality was expected and curiosity, although evoked at the time of marriage, was not addressed. Instead they learned that talking about sexuality and having questions about married life was a sign of having no shame.

*"I am looking forward to my marriage and I want to ask questions but I do not talk about this with my mother.... she doesn't even know I menstruate. How can we talk about these things?" *(19 year old)

The young women either lacked or had deficient knowledge about sexuality, contraception and pregnancy. They knew marriage included some kind of physical contact with the husband but could neither explain nor understand the actual sexual encounter. When asked how a woman conceived a child they responded that it happened after marriage and at God's will.

*"My sister is happy. Her husband is a plumber. My elder sister used to say that she has had enough children but then she had another son... now she says the same thing. It's your fate as to how many children you will have and God's Will!" *(22 years old)

#### Finding cracks in the wall of silence

The young women interviewed knew about contraceptives through television (TV) advertisements, but they lacked full understanding of how to use and access them. The mothers approved of their daughters having contact with older cousins, sisters and aunts as a source of information about sexuality and childbearing near the time of their marriage. The information however was vague and not fully understood by the young women. There were a limited number of young women who sought out or accepted "pockets to think," which we define as space and time approved by their mothers to talk with their fiancés prior to marriage. Those young women communicated with their fiancés briefly about general issues using mobile phones. These conversations sparked the women's own thinking about planning for their married life and the number of children they want to have, although this thinking was not shared with others. This small sub-group was keen to learn about child spacing and accessibility to contraceptives.

#### Security lies in obedience

The main theme identified from the perspectives women shared in the interviews and FGDs as the underlying meaning of their lives well illustrates the feelings and experiences of the young women regarding their upcoming marriage: security lies in obedience. They claimed to love and trust their parents, listening to their opinions and feeling secure in following their rules as they were older and more knowledgeable. A "good daughter" was defined as one who abided by the rules. The young women trusted that continued family support would ensure security in future life.

*"I love my mother. If she is happy so am I. I know that my parents know what is best for me as they are older and wiser. If they think I should marry this person then I am fine."*(17 years old)

Participants expressed fear of the consequences of not following the rules.

*"If one decides oneself on whom to marry, then one does not have the support of the parents. You are bound to like the husband chosen for you by your parents. If one decides oneself and does not like the husband later then parents say it was your choice and you lose ..... you are alone......have no one to turn to and no support from the family and no security anywhere." *(FGD 1)

## Discussion

In the traditional Pakistani society, marriage is seen as a family, communal and societal affair more than a joint enterprise of the couple. Girls are socialised from childhood into the role of a wife who should fulfil the expectations of the mother-in-law and husband and who the parents were proud of handing over to the new family [18,19]. This was brought out also in the earlier study by the authors, which interviewed married young women. It showed how the women were raised by parents, family and broader society to practice obedience in silence and to not question the decisions of elders, first in their parents' homes and then in their new homes after marriage [12]. The unmarried participants in the current study affirm the perspectives of the married women in the earlier study, sharing in detail how the foundation for the ideals of submissiveness, obedience and silence is laid.

### Socialized towards family

Participants were brought up in a social environment in which adolescent girls were neither expected nor allowed to move on their own outside the home or to meet young men. Instead they were busy in the home learning household chores and helping their mothers, who underscored the need to learn *sabar *(patience) and to trust the decisions of the elders, including about their own health and fertility. The message of *sabar *that was forcefully reiterated from childhood prepared them for the coming challenges of a minimal voice in the decisions around marriage. These findings are in accordance with the Pakistani tradition where collective welfare outweighs individual well-being. Only after considering all other family members needs are women allowed to think about their own [20]. Participants learned that "being acceptable" in society's eyes meant becoming selfless. This limited self-concept is also described by Kagitcibasi, who illustrated how children in collectivist societies in Turkey lack an understanding of self, as a concept of a person with her or his own desires, preferences, attributes and abilities [21]. In our study the young women defined their self-identity in relation to the community and family, especially the parents, which is consistent with other findings from South Asia [22]. A study by Wilson-Williams et al (2008) on violence against women in India describes the same socialization into submissiveness and obedience. The women expressed that their husbands felt that wives should always obey them and failure to do so resulted in their being abused and asked to leave home [23]. Our earlier study on young married women illustrates how the submissiveness they are trained into from childhood results in lack of control over their life [12].

### Remaining in the comfort zone until the time of marriage

At the time of puberty and first menstruation the young women learned to keep quiet about women's issues and avoided thinking about marriage, sexual relations and childbearing, about which they had little knowledge. Instead they remained in their comfort zones, postponing thinking about such issues until the time of marriage. Living in an illusion of being safe by not admitting their own desires and ambitions, the young women did not realise their lack of control over their lives. This false perception of safety, comfort and control was supported by their families, who curtailed their mobility and ensured few external exposures. These findings are supported by studies in Pakistan and India, where women reported that they had limited control over their lives and that decisions pertaining to their pregnancy were taken by others [18,24,25]. Likewise, Mathur et al (2001) showed that Nepali adolescent girls were unable to realize their hopes for continued education, better paid jobs and delayed marriage and childbearing because of restrictive social norms and institutions [26]. This is also consistent with other studies identifying the need for accurate information about sexuality, reproduction and contraception in South Asian adolescents [2,9,12,21,26].

### Role of media

In our study the young women referred to TV advertisements as a source of information on contraception. Television played the role of a peer in the participants' lives, as a source of both entertainment and education. A study in Pakistan found that men had access to different types of media while young women had limited access because of their restricted mobility and fewer opportunities from which to choose themselves [7]. Participants also referred to TV serials as showing importance given to the husband and his family which evoked their fears about lack of control over their future lives. The young women identified and recognised how the roles in society affected the lives of women and caused them to question societal norms and wonder about their future. Some parents allowed the young women to communicate with young married cousins and/or their fiancés during the engagement period. They started to discuss married life and their approaching marriage triggered their thinking. They were keen to learn more about reproductive health issues and hoped to be able to communicate with their future husbands.

### Methodological considerations

In this study, FGDs followed individual interviews. While the interviews focussed on individual concerns, the FGDs revolved around concerns about young women in general, and proved to be a successful strategy for further exploring feelings towards marriage and adulthood. They proved to be a valuable complement to the individual interviews as they allowed the participants to freely carry out lively discussions and bring up issues pertaining to their friends and acquaintances. Allowing the young women to choose the venue for the interviews created a favourable environment for discussions, which provided a safe, comfortable forum in which they were able to openly voice their thoughts and feelings about growing up and entering into marriage.

A limitation of the study could be that the participants might have had to report to their parents and therefore preferred not to answer certain questions. They could even have been instructed by their mothers between the first and second interview.

The findings of this study are based on a limited number of interviews and focus group discussions. They can thus not be generalised, but it is plausible to think that what the participants shared is also valid for other young women from similar backgrounds and in similar situations.

## Conclusions

The experiences, views and fears of the young women in this study demonstrate how their socialization into submissiveness in a culture of silence around sexuality, lays the foundation for lack of control over their future reproductive health. This study identifies the role of television as a peer in their lives and highlights the need to bring about attitudinal changes within the home and extended family environment so that young women's confusion and needs regarding sexuality can be addressed prior to marriage. Since these young women are allowed to move in a social network that is judged as acceptable by the family, community- based initiatives could provide socially acceptable informal discussion groups where young women could meet to discuss sexuality, child bearing and other marriage-related issues and have their queries addressed. These discussion groups and other future innovative initiatives, which are based on an understanding of the young women's needs and have community acceptability, are much-needed and will be useful. Uplifting women's self-identity and integrating women into decision-making, first with their parents and later with their husbands, must be recognised and promoted at all levels in society and integrated into these initiatives. The results of this study are important for public health planning in Pakistan and for those working on women's health issues nationally and internationally.

## Competing interests

The authors declare that they have no competing interests.

## Authors' contributions

SH was the main author of the manuscript and involved in all aspects of the study. EJ and BR provided scientific oversight and feedback throughout the development of the study and manuscript. All co-authors have seen and approved the final version of the paper and have agreed to its submission for publication.

## Pre-publication history

The pre-publication history for this paper can be accessed here:

http://www.biomedcentral.com/1471-2458/10/164/prepub
